# Hæmaturia in Renal Tuberculosis

**Published:** 1910-05-07

**Authors:** 


### Medicine.

### HÆMATURIA IN RENAL TUBERCULOSIS.

Not long since we laid stress upon the importance of recognising the possibility or even likelihood of renal tuberculosis when, in young subjects, hæmaturia develops without apparent cause, and without any other symptoms at first. Hæmaturia may be the very earliest indication of tuberculous disease of the kidney. The following is a case in point. The patient was a woman twenty-eight years of age, who came into hospital after she had suffered from abundant hæmaturia on and off for sixteen months. There was nothing in her family or personal histories to attract attention, and the patient had been in the best of health hitherto, except that she had been very subject to coughs and colds in the winter time.

In December 1905 she had her first attacks of hæmaturia. On each occasion the urine was the colour of blood from beginning to end of micturition, but the attacks did not disturb her much, for there was no pain, no frequency of micturition, and they disappeared in about four days almost as suddenly as they came. After recurring repeatedly during December 1905 and January 1906, they ceased altogether for two months; but in March 1906 they recurred once more, and from that time until May 1907 the urine was never free from blood, though it was sometimes almost clear, and sometimes distinctly red.

These attacks of hæmaturia, which constitute a dominant feature in the case, were all spontaneous, capricious, and yet persistent, and the urine was always coloured equally throughout. Resistant to all kinds of medicinal treatment, the attacks were often so marked that the patient herself thought she was passing pure blood. This repetition and this abundance of hæmaturia gradually led to loss of strength and colour, so that the patient decreased in weight and became lacking in energy and profoundly anæmic.

In August 1906, nine months after the first onset, frequency of micturition, especially at night, began to attract attention. In January 1907, in addition to hæmaturia and nocturnal frequency of micturition, pus was first obvious to macroscopic examination; large quantities were passed, attracting the patient's own notice. It was not until February 1907 that pain was first complained of; even then it was not severe, being limited to a sense of discomfort in the hypogastric region when the urine was not soon voided, and later a dull ache in the left loin, radiating downwards and forwards towards the bladder.

Tubercle bacilli were found in the centrifugalised urinary deposit in abundance, and the diagnosis was clear. The only question was whether to continue solely with medicinal treatment or whether to adopt surgical measures for the removal of the left kidney. There is no need to go into the pros and cons of this here, for the main point that the case is intended to illustrate is the importance of hæmaturia as the earliest symptom of the mischief in some cases.

Suffice it to say that an operation was performed, and the left kidney was found to be extensively affected by ulcerating caseous deposits of tubercular material. So far, the result has been good; but there is reason to suppose that the mischief was not confined to the kidney, but had spread to the left ureter and the bladder. It is possible that the patient may be able to heal the lesions in the latter now that there are no longer continual fresh supplies of bacilli coming down from the left kidney, but there is a great danger that the bacillary infection may extend up the right ureter and involve the right kidney. The macroscopic hæmaturia ceased from the time that the operation was performed.

